# Combination of proton-pump inhibitor and anti-CD3 F(ab’)_2_ for islet neogenesis and immune modulation in type 1 diabetes

**DOI:** 10.1093/immadv/ltag017

**Published:** 2026-07-22

**Authors:** Tung-Yun Wu, Che-Yi Chen, Michael Chen, Chang-Hung Wang, An-Pei Kao, Kai-Hung Wang, Kuo-Hsiang Chuang, Shyr-Yi Lin

**Affiliations:** Graduate Institute of Pharmacognosy, Taipei Medical University, Taipei, Taiwan; Ph.D. Program in Clinical Drug Development of Herbal Medicine, Taipei Medical University, Taipei, Taiwan; Graduate Institute of Pharmacognosy, Taipei Medical University, Taipei, Taiwan; Graduate Institute of Pharmacognosy, Taipei Medical University, Taipei, Taiwan; Cytoarm Co., Ltd, Taipei, Taiwan; Department of Obstetrics and Gynecology, Kuo General Hospital, Tainan City, Taiwan; Center for Reproductive Medicine, Kuo General Hospital, Tainan, Taiwan; Graduate Institute of Pharmacognosy, Taipei Medical University, Taipei, Taiwan; Ph.D. Program in Clinical Drug Development of Herbal Medicine, Taipei Medical University, Taipei, Taiwan; Master Program for Clinical Pharmacogenomics and Pharmacoproteomics, Taipei Medical University, Taipei, Taiwan; Traditional Herbal Medicine Research Center, Taipei Medical University Hospital, Taipei, Taiwan; Division of Gastroenterology, Department of Internal Medicine, Taipei Medical University Hospital, Taipei, Taiwan

**Keywords:** anti-CD3 F(ab’)_2_, proton-pump inhibitor, type 1 diabetes, autoimmune disease, islet neogenesis

## Abstract

**Introduction:**

Type 1 diabetes (T1D) is an autoimmune disease characterized by T cell–mediated destruction of pancreatic β cells. Current insulin therapy does not address autoimmune pathology or restore endogenous β-cell function, highlighting the need for disease-modifying strategies.

**Methods:**

We developed a combination therapy integrating a proton pump inhibitor (PPI; omeprazole) with a non–Fc-binding anti-CD3 F(ab’)_2_ antibody fragment (anti-CD3 F(ab’)_2_) to concurrently promote β-cell neogenesis and modulate autoimmune T-cell responses. T1D was induced in nonobese diabetic (NOD) mice using a cyclophosphamide-accelerated protocol (CY-NOD). Following hyperglycemia onset, mice received combination treatment and were evaluated by blood glucose monitoring, flow cytometric analysis of T-cell populations, and assessment of pancreatic β-cell function. Therapeutic efficacy was further examined in spontaneous-onset NOD mice.

**Results:**

Combination therapy restored normoglycemia without exogenous insulin, reduced lymphocytic infiltration in pancreatic islets, and preserved endogenous insulin secretion. Treatment decreased pathogenic CD4^+^ and CD8^+^ T cells, increased regulatory T cells, and enhanced transforming growth factor-β expression. Comparable and durable glycemic control was observed in spontaneous-onset NOD mice.

**Conclusions:**

Concurrent modulation of β-cell function and autoimmune immunity achieved sustained glycemic control in T1D models. The use of clinically approved agents supports the translational potential of this non–insulin-dependent therapeutic strategy.

## Graphical Abstract

**Graphical Abstract:**
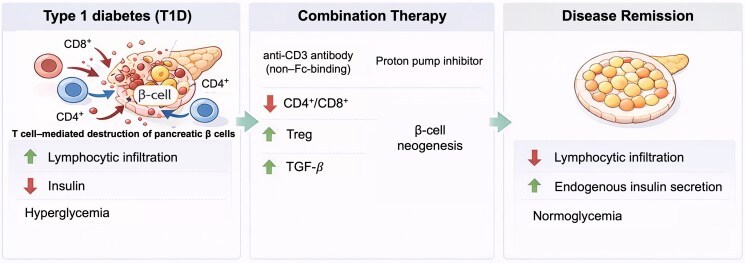

## Introduction

Type 1 diabetes (T1D) is an autoimmune disease driven by immune-mediated destruction of pancreatic islets, involving both innate and adaptive immune responses [1, 2]. Among these, CD8^+^ and CD4^+^ T cells play central roles in β-cell destruction. Upon activation through the major histocompatibility complex (MHC)–T cell receptor (TCR) pathway, autoreactive T cells infiltrate pancreatic islets and induce β-cell death via the secretion of perforin, granzymes, and proinflammatory cytokines, including interferon-γ (IFN-γ) and tumor necrosis factor-α (TNF-α) [3, 4]. Progressive β-cell loss results in insulin deficiency, and patients transition through normoglycemic, presymptomatic dysglycemic, and symptomatic stages of disease [5, 6].

Insulin supplementation combined with dietary management remains the cornerstone of T1D treatment and is required lifelong. However, insulin therapy does not address the underlying autoimmune pathology or halt disease progression. As a consequence, within 5 years of diagnosis, substantial proportions of patients develop chronic complications, including neuropathy (21.8%), nephropathy (13.1%), retinopathy (12.2%), and cardiovascular disease (19.2%), which collectively contribute to T1D-associated mortality [7, 8]. Moreover, intensive insulin administration is associated with adverse effects such as weight gain, hypoglycemia, and ketoacidosis, further increasing morbidity and mortality risks [3, 9]. These limitations have driven substantial efforts toward therapeutic strategies aimed at preserving pancreatic islet function [10].

Two principal approaches have emerged for β-cell preservation in T1D: immune modulation and stimulation of islet regeneration. Anti-CD3 antibodies were initially developed to prevent allograft rejection [11] and were later shown to preserve β-cell function in T1D [12, 13]. CD3 forms a critical component of the TCR complex and is essential for T-cell activation [14]. Importantly, Fc-mediated binding of anti-CD3 antibodies has been associated with adverse effects. To overcome this limitation, non-Fc–binding anti-CD3 antibodies were developed, and Chatenoud *et al*. demonstrated their ability to reverse new-onset diabetes in nonobese diabetic (NOD) mice [15]. Mechanistically, anti-CD3 F(ab’)_2_ engages the CD3 component of the T-cell receptor complex and modulates TCR-mediated signaling without Fc receptor–dependent crosslinking. This property is important because Fc receptor engagement by conventional anti-CD3 antibodies can induce excessive T-cell activation and cytokine release [16, 17]. In contrast, anti-CD3 F(ab’)_2_ reduces pathogenic autoreactive T-cell responses while favoring immune regulatory pathways, including the expansion of Foxp3^+^ regulatory T cells and induction of a TGF-β–associated tolerogenic environment [18–20]. These immunomodulatory effects limit autoimmune β-cell destruction and create a permissive immune milieu for β-cell preservation or recovery. Subsequent studies revealed that anti-CD3 therapy selectively depletes pathogenic autoreactive T cells while promoting the expansion of regulatory T cells (Tregs), thereby attenuating autoimmune β-cell destruction [19, 21, 22].

Clinically, non-Fc–binding anti-CD3 antibodies (anti-CD3 F(ab’)_2_), including otelixizumab and teplizumab, have demonstrated efficacy in patients with recent-onset T1D [23–25]. In a phase II trial, teplizumab significantly delayed disease onset in high-risk, autoantibody-positive individuals with preserved insulin secretion [26–28]. Nevertheless, progression to overt diabetes ultimately occurred, potentially reflecting insufficient β-cell regeneration. Furthermore, in patients with established T1D and minimal residual β-cell mass, anti-CD3 therapy alone has shown limited efficacy [29].

A complementary strategy focuses on promoting β-cell regeneration. Proton pump inhibitors (PPIs), such as omeprazole and pantoprazole, are widely prescribed for gastroesophageal reflux disease (GERD) [30]. In rodent models, PPIs have been shown to induce β-cell neogenesis through increased gastrin secretion from gastric G cells, with gastrin acting as a β-cell growth factor [31, 32]. In a phase II study in patients with type 2 diabetes, pantoprazole increased circulating gastrin and insulin levels. However, clinical studies in T1D have not demonstrated significant increases in β-cell mass, despite elevated markers of β-cell proliferation [33], suggesting that newly generated β-cells may be eliminated by ongoing autoimmune attack.

In the present study, we hypothesized that simultaneous immune modulation and stimulation of β-cell regeneration would be required to preserve islet function in early-stage T1D. To test this concept, we employed a cyclophosphamide-accelerated NOD (CY-NOD) mouse model, which enables controlled induction of hyperglycemia and facilitates evaluation of immune modulation and β-cell preservation. A non-Fc-binding anti-CD3 F(ab’)_2_ antibody fragment (clone 145-2C11) was used to modulate autoimmune T-cell activity and was combined with a proton pump inhibitor (PPI; omeprazole) previously reported to support β-cell recovery [32, 34].

This dual-target strategy was designed to achieve immune regulation while maintaining islet integrity. To assess its translational relevance, both accelerated and spontaneous-onset NOD mouse models were used to evaluate glycemic control, T-cell dynamics, and β-cell function following combination therapy. Given that both agents are approved for clinical use, this approach represents a promising non–insulin-dependent therapeutic strategy with potential to improve glycemic control and delay disease progression in T1D.

## Materials and methods

### Reagents

Cyclophosphamide (CY; CAS No. 50-18-0) was purchased from Molnova (Ann Arbor, MI, USA). Omeprazole (Lometin®; China Chemical & Pharmaceutical Co., Ltd., Taipei, Taiwan). Hamster anti-mouse CD3ε F(ab’)_2_ antibody fragments (clone 145-2C11; BE0001-1FAB) were purchased from Bio X Cell (West Lebanon, NH, USA). Goat anti-mouse IgG Fcγ–horseradish peroxidase (HRP) was obtained from Jackson ImmunoResearch (West Grove, PA, USA). Dextrose (CAS No. 50-99-7) was purchased from Sigma-Aldrich (St. Louis, MO, USA), and isoflurane was obtained from Attane (Bethlehem, PA, USA). Flow-cytometry antibodies, including anti-CD4–FITC (11-0042), anti-CD8–FITC (11-0081), anti-CD3–PE (12-0031), and anti-Foxp3–PE (12-5773), were purchased from eBioscience (San Diego, CA, USA). Phosphate-buffered saline (PBS) was purchased from Invitrogen (Carlsbad, CA, USA).

### Mice

Female NOD/ShiLtJNarl (NOD) mice were purchased from the National Laboratory Animal Center, Taiwan (NLAC), and housed under specific-pathogen-free (SPF) conditions with a 12-h light/dark cycle. Sterile water and standard chow were provided *ad libitum*.

### Diabetes induction, monitoring, and definitions

#### CY-accelerated model (CY-NOD)

NOD mice received a single intraperitoneal injection of cyclophosphamide (CY; 300 mg/kg) on day 0. Blood glucose was monitored every 3 days after CY administration.

#### Spontaneous-onset model

NOD mice were monitored longitudinally until spontaneous-onset of hyperglycemia.

For both models, blood glucose was measured using tail-vein sampling with a Roche ACCU-CHEK Performa glucometer (USA). Diabetes onset was defined as blood glucose >300 mg/dL for more than 2 consecutive days, and remission was defined as blood glucose <300 mg/dL.

### Treatment with anti-CD3 F(ab’)_2_ and proton pump inhibitor

In the CY-NOD model, NOD mice received intravenous injections of anti-CD3 F(ab’)_2_ (10 µg per mouse) on days 12–16, 22, and 32 after CY injection. In addition, mice were administered the PPI omeprazole (40 mg/kg/day) by oral gavage starting on day 12 and continuing until the end of the treatment period.

In the spontaneous-onset model, treatment was initiated upon confirmation of diabetes onset (as defined above), using the same anti-CD3 F(ab’)_2_ and omeprazole regimen.

### ELISA

Mouse plasma was collected and analyzed according to the manufacturers’ instructions. C-peptide was quantified using the mouse C-peptide ELISA kit (Elabscience, E-EL-M0354), and TGF-β1 was quantified using the TGF-β1 Human/Mouse Uncoated ELISA Kit (Invitrogen, 88-8350-22). Briefly, plasma samples were added to capture antibody–coated plates, followed by biotinylated detection antibody and streptavidin–HRP. The reaction was developed using TMB substrate, stopped with 2 N H_2_SO_4_, and absorbance was measured at 450 nm.

### Flow cytometry

Mice were euthanized under isoflurane anesthesia. Peripheral splenocytes were collected, and red blood cells were lysed using ammonium–chloride–potassium (ACK) lysis buffer (Thermo Fisher Scientific, A1049201). Cells were stained with anti-CD4–FITC, anti-CD8–FITC, anti-CD3–PE, and anti-Foxp3–PE. Intracellular Foxp3 staining was performed using the eBioscience™ Foxp3/Transcription Factor Staining Buffer Set according to the manufacturer's protocol. Samples were analyzed on a Sony SA3800 flow cytometer.

### Immunohistochemistry for β-cell function and neogenesis

Paraffin-embedded pancreas sections were immunostained for insulin and cytokeratin-19 (CK-19). Antigen retrieval was performed in 0.1 M citrate buffer (pH 6.0). After blocking with 10% goat serum and 1% bovine serum albumin (BSA) in PBS containing Tween-20 (PBST), sections were incubated with primary antibodies (anti-insulin or anti-CK-19), followed by HRP-conjugated secondary antibodies. Signals were developed using 3,3′-diaminobenzidine (DAB) and counterstained with hematoxylin. Hematoxylin and eosin (H&E) staining was performed for morphological assessment. Histological images were acquired and analyzed using an ImageScope system. Insulin and CK-19 staining signals were quantified using ImageJ software and expressed as staining intensity normalized to the corresponding islet area (intensity/islet area, μm^2^).

### Statistical analysis

Statistical analyses were performed using SPSS. *P* < 0.05 was considered statistically significant. Group comparisons were performed using one-way analysis of variance (ANOVA) and paired *t*-tests, as appropriate.

## Results

### Combination of anti-CD3 F(ab’)_2_ and PPI therapy reverses diabetes progression in cyclophosphamide-accelerated NOD mice

**Figure 1:**
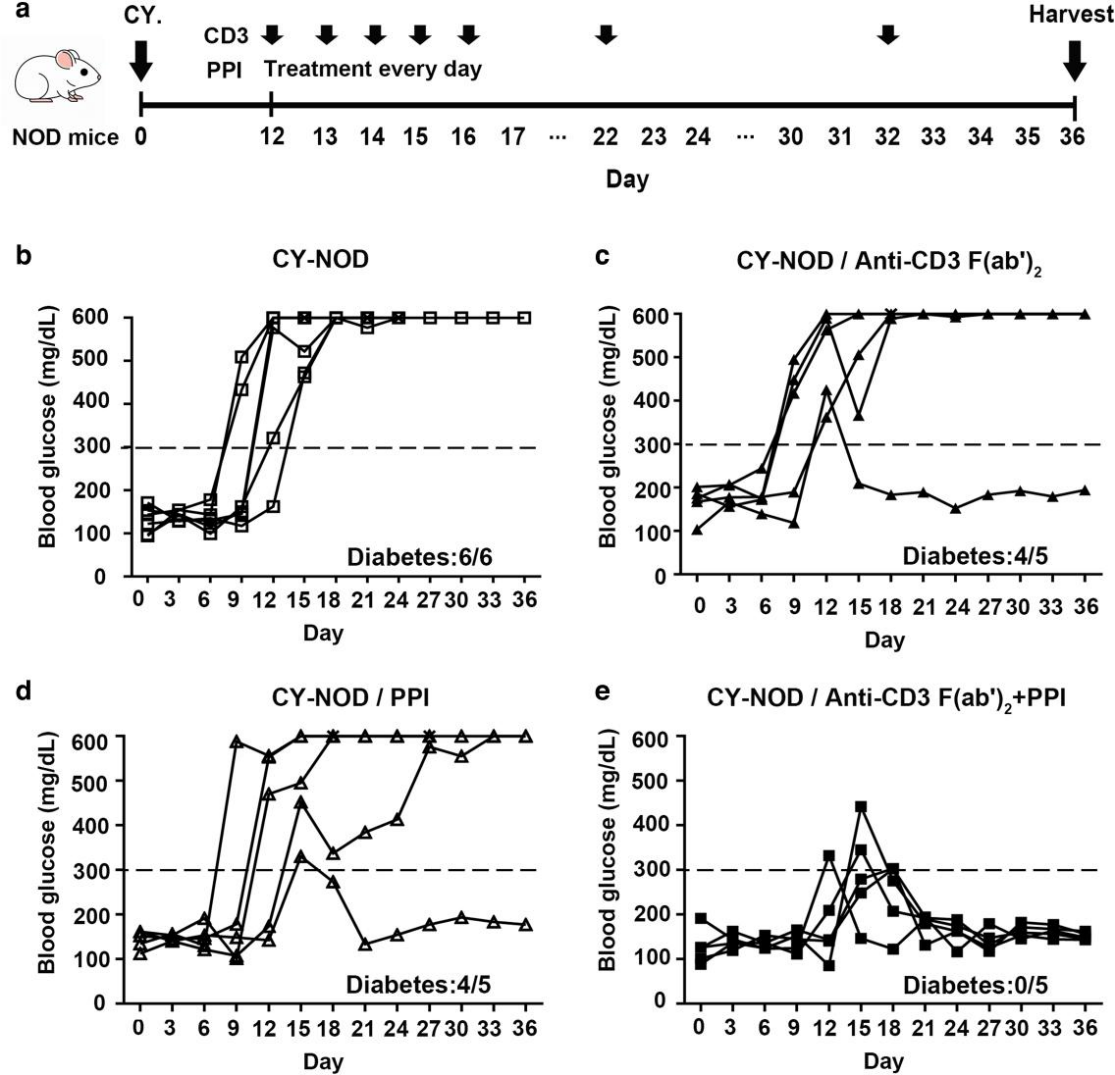
Blood glucose monitoring and serum C-peptide in CY-NOD mice treated with anti-CD3 F(ab’)_2_ and/or PPI. (a) Treatment schedule. Fifteen-week-old female NOD mice received a single intraperitoneal injection of cyclophosphamide (CY; 300 mg/kg) to accelerate diabetes onset, followed by treatment with anti-CD3 F(ab’)_2_ (clone 145-2C11; 10 µg/mouse, intravenous) and/or omeprazole (40 mg/kg/day, oral gavage). Blood glucose was monitored, and diabetes onset was defined as blood glucose >300 mg/dL for >2 consecutive days. (b–e) Blood glucose profiles in (b) untreated diabetic controls (*n* = 6), (c) anti-CD3 F(ab’)_2_ monotherapy (*n* = 5), (d) omeprazole monotherapy (*n* = 5), and (e) combination therapy (*n* = 5).

Nonobese diabetic (NOD) mice are a well-established animal model of type 1 diabetes, with ∼60% of female mice spontaneously developing diabetes between 8 and 26 weeks of age. To accelerate disease onset, 15-week-old NOD mice received a single injection of cyclophosphamide (CY), generating a cyclophosphamide-accelerated NOD (CY-NOD) model. Starting 12 days after CY administration, NOD mice were treated with an anti-CD3 F(ab’)_2_ administered intravenously and a PPI administered orally, either as monotherapies or in combination, alongside control treatments (Fig. 1a). In the control group, all CY-NOD mice developed hyperglycemia within 9–15 days following CY injection and remained severely hyperglycemic until death, accompanied by marked body weight loss exceeding 20% (Fig. 1b; Supplementary Figure S1a). Treatment with either anti-CD3 F(ab’)_2_ alone or PPI alone resulted in disease remission in 1 of 5 mice in each group (Fig. 1c and d). Although anti-CD3 F(ab’)_2_ monotherapy did not induce complete diabetes remission in all hyperglycemic mice, it significantly reduced body weight loss and mortality compared with untreated controls, indicating a disease progression–delaying effect (Supplementary Figure S1b and c). In contrast, all diabetic CY-NOD mice receiving combination anti-CD3 antibody and PPI therapy reverted to normoglycemia and maintained stable glycemic control throughout the observation period (Fig. 1e). Notably, no mortality was observed in this treatment group, and body weight was maintained without significant loss during the course of therapy (Supplementary Figure S1d). Collectively, these findings demonstrate that combination anti-CD3 F(ab’)_2_ and PPI therapy effectively reverses diabetes progression and provides durable glycemic control in CY-NOD mice.

### Combination therapy modulates immune responses and preserves insulin secretory function

**Figure 2:**
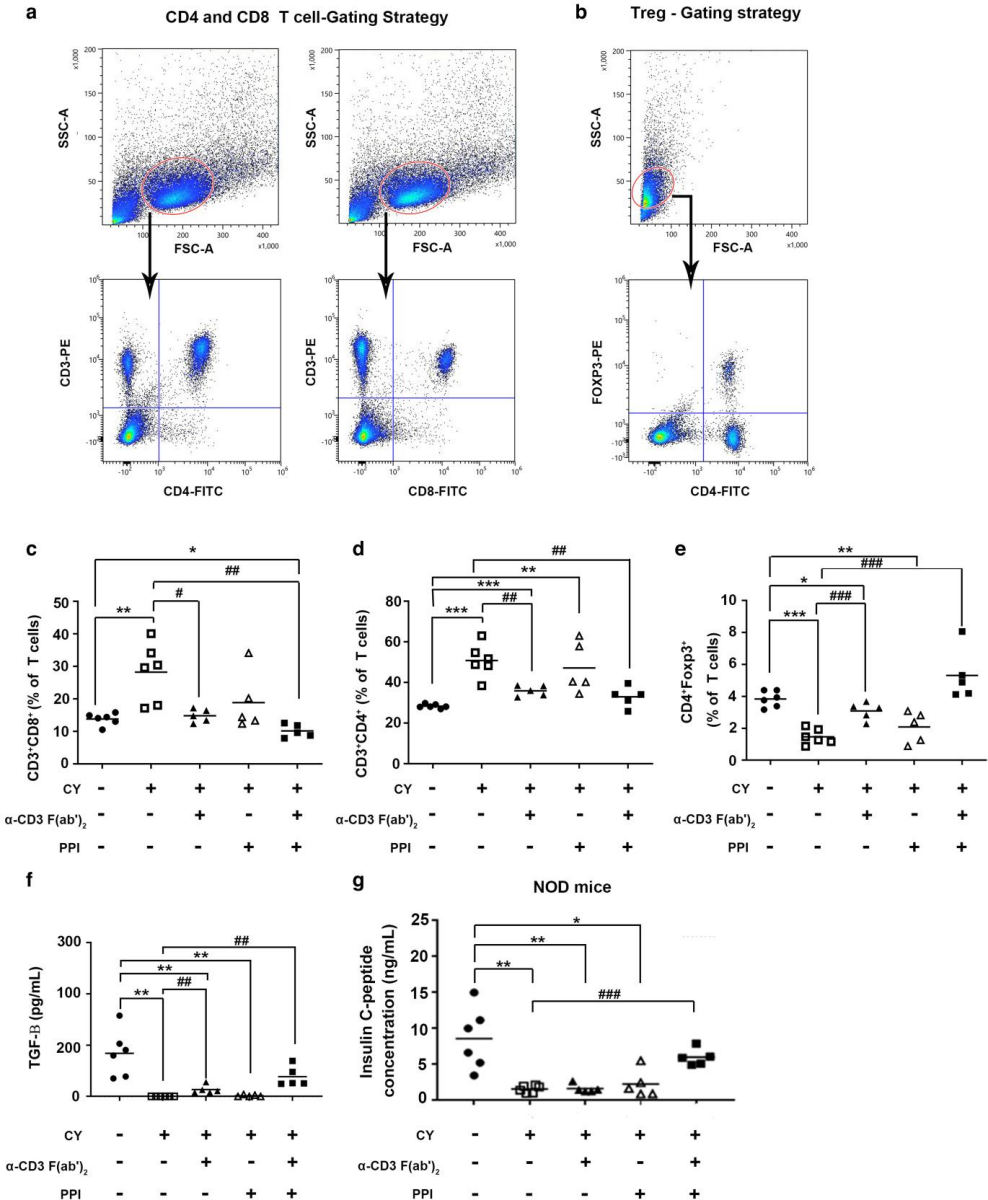
Splenic T-cell populations and serum TGF-β in CY-NOD mice treated with anti-CD3 F(ab’)_2_ and/or PPI. CY-NOD mice were treated with anti-CD3 F(ab’)_2_ (10 µg/mouse, intravenous) and/or omeprazole (40 mg/kg/day, oral gavage). Mice that survived to Day 36 after treatment were included for downstream analyses (euglycemic, *n* = 6; untreated, *n* = 6; anti-CD3 F(ab’)_2_ monotherapy, *n* = 5; PPI monotherapy, *n* = 5; combination, *n* = 5). Splenocytes were collected after treatment and analyzed by flow cytometry. Representative gating strategies are shown for (a) CD4^+^ and CD8^+^ T cell identification and (b) regulatory T cell (Treg) identification. Quantification of (c) CD3^+^CD8^+^ T cells, (d) CD3^+^CD4^+^ T cells, and (e) regulatory T cells (Treg; CD4^+^Foxp3^+^) across treatment groups. (f) Serum TGF-β and (g) C-peptide were measured by ELISA. *P < 0.05, **P < 0.01, ***P < 0.001 vs euglycemic mice; ^#^*P* < 0.05, ^##^*P* < 0.01, ^###^*P* < 0.001 vs untreated diabetic controls.

Previous studies have reported that anti-CD3 F(ab’)_2_ has been reported to suppress effector T-cell responses and promote immunoregulatory cell populations, particularly regulatory T cells (Tregs) [20], thereby limiting autoimmune-mediated β-cell destruction. Accordingly, we examined systemic immune modulation by profiling T-cell subsets in splenic lymphocytes (Fig. 2a and b). Analysis of splenic lymphocytes showed that anti-CD3 F(ab’)_2_ treatment, either as monotherapy or in combination with the PPI, significantly reduced the frequencies of CD3^+^CD8^+^ and CD3^+^CD4^+^ T cells compared with untreated CY-NOD controls (Fig. 2c and d), whereas PPI monotherapy did not significantly change these effector T-cell populations. In addition, anti-CD3 F(ab’)_2_ treatment increased the splenic Treg population (Fig. 2e), with the most pronounced expansion observed in the combination therapy group, while PPI monotherapy did not significantly alter splenic Treg frequency under the conditions tested. Because immune tolerance can involve both Treg-associated pathways and transforming growth factor-β (TGF-β) signaling, we further quantified circulating TGF-β levels. Figure 2f shows that, in untreated and PPI-treated diabetic CY-NOD mice, serum TGF-β levels were below the assay detection limit (16 pg/mL), markedly lower than those observed in untreated euglycemic mice (84.15 ± 18.52 pg/mL). Anti-CD3 F(ab’)_2_ monotherapy yielded low TGF-β levels (13.47 ± 3.751 pg/mL), whereas combination treatment increased serum TGF-β to a clearly detectable level (38.64 ± 8.859 pg/mL). Collectively, these results indicate that anti-CD3 F(ab’)_2_ treatment is the primary driver of effector T-cell reduction and Treg expansion, whereas PPI monotherapy does not significantly alter T-cell or Treg populations under the conditions tested. Notably, the combination regimen was associated with the most pronounced immunoregulatory profile, characterized by reduced effector T-cell frequencies, increased Treg frequencies, and elevated circulating TGF-β.

To examine whether combination therapy preserved insulin secretory function, serum was collected and insulin C-peptide concentrations were measured. Compared with untreated euglycemic mice (7.611 ± 1.729 ng/mL), C-peptide levels in untreated CY-NOD mice were markedly reduced (1.664 ± 0.252 ng/mL). Neither anti-CD3 antibody monotherapy (1.599 ± 0.186 ng/mL) nor PPI monotherapy (2.347 ± 0.602 ng/mL) significantly increased C-peptide concentrations. In contrast, combination anti-CD3 antibody and PPI therapy increased serum C-peptide levels by approximately four-fold (6.679 ± 1.343 ng/mL) relative to untreated CY-NOD controls, reaching levels comparable to those observed in untreated euglycemic mice (Fig. 2g). Collectively, these results indicate that combination anti-CD3 F(ab’)_2_ and PPI therapy not only modulates pathogenic immune responses but also preserves endogenous insulin secretory capacity in CY-NOD mice.

### Combination therapy reduces islet immune cell infiltration and promotes β-cell neogenesis

**Figure 3:**
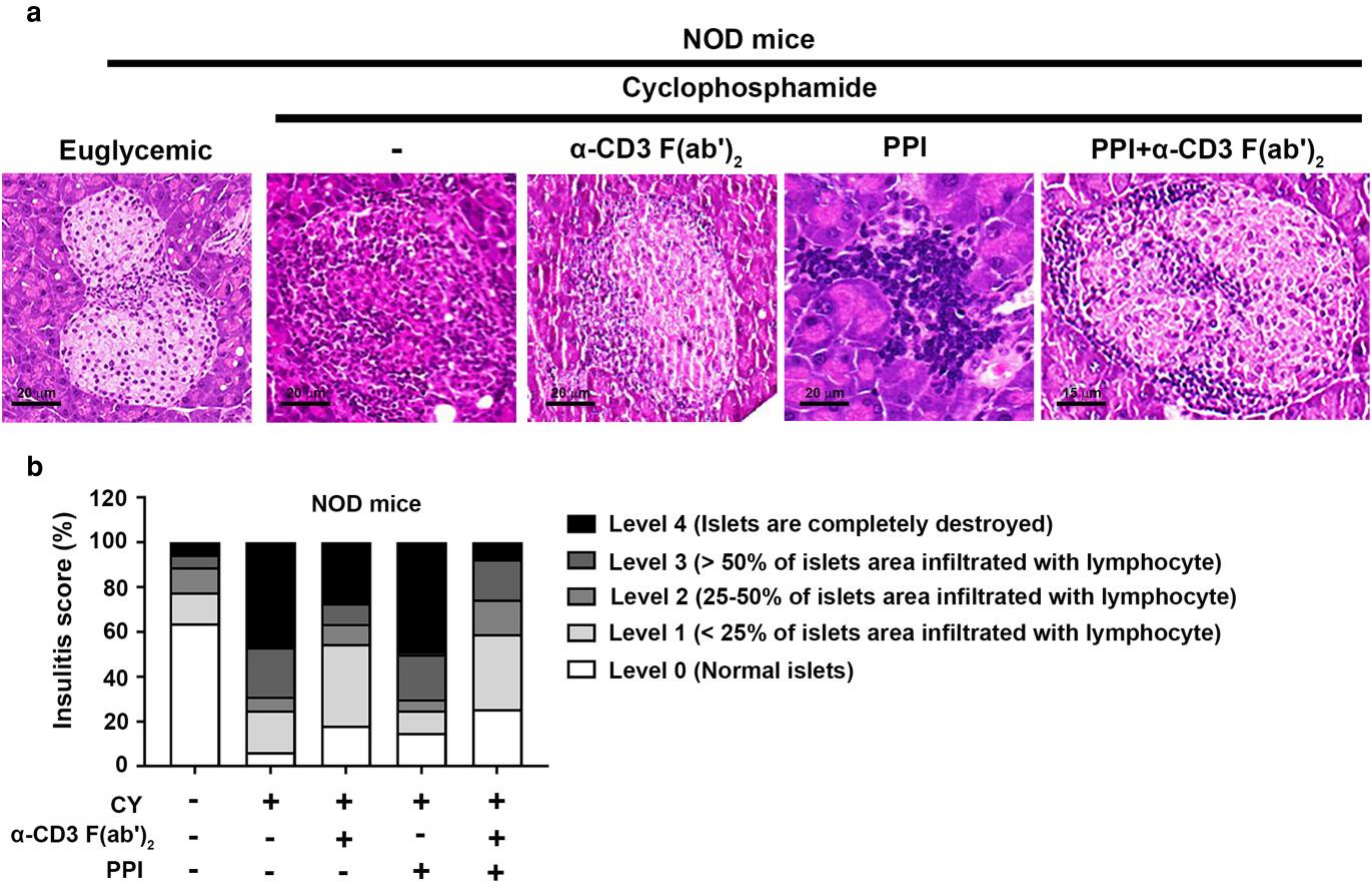
Histological assessment of insulitis severity after anti-CD3 F(ab’)_2_ and/or PPI treatment. At the end of the experiment (day 36), pancreatic tissues were collected from each group (euglycemic controls, *n* = 6; untreated diabetic controls, *n* = 6; PPI monotherapy, *n* = 5; anti-CD3 F(ab’)_2_ monotherapy, *n* = 5; combination therapy, *n* = 5), fixed in 4% paraformaldehyde, paraffin-embedded, and stained with H&E to evaluate lymphocytic infiltration of islets. (a) Representative micrographs showing insulitis across treatment groups. (b) Quantification of insulitis severity. A total of 11–39 islet cross-sections per group were scored. Islets were graded as: level 0, no infiltration; level 1, <25% infiltrated; level 2, 25–50%; level 3, >50%; level 4, islet largely destroyed.

Anti-CD3 F(ab’)_2_–containing regimens have been reported to modulate autoimmunity through induction of regulatory T cells (Tregs) and TGF-β–associated immunoregulation [35, 36]. To evaluate the histopathological impact of anti-CD3 F(ab’)_2_ and PPI combination therapy, we scored insulitis severity using a five-level scale (levels 0–4) and grouped the results into three categories: no infiltration (level 0), mild insulitis (levels 1–2), and severe insulitis (levels 3–4). Four weeks after cyclophosphamide (CY) injection, most islets in the diabetic control group exhibited severe immune cell infiltration (21.88% at level 3 and 46.8% at level 4), consistent with extensive insulitis on H&E staining. A similar distribution was observed in the PPI monotherapy group (20% at level 3 and 50% at level 4), indicating minimal improvement in insulitis severity with PPI alone. In contrast, anti-CD3 F(ab’)_2_ monotherapy and combination therapy preserved islet architecture, with more than 50% of islets remaining in the no-infiltration or mild insulitis categories (levels 0–2). Although some islets still showed infiltration—likely because treatment was initiated after diabetes onset—anti-CD3–based regimens reduced the proportion of severely infiltrated islets (Fig. 3a and b).

**Figure 4:**
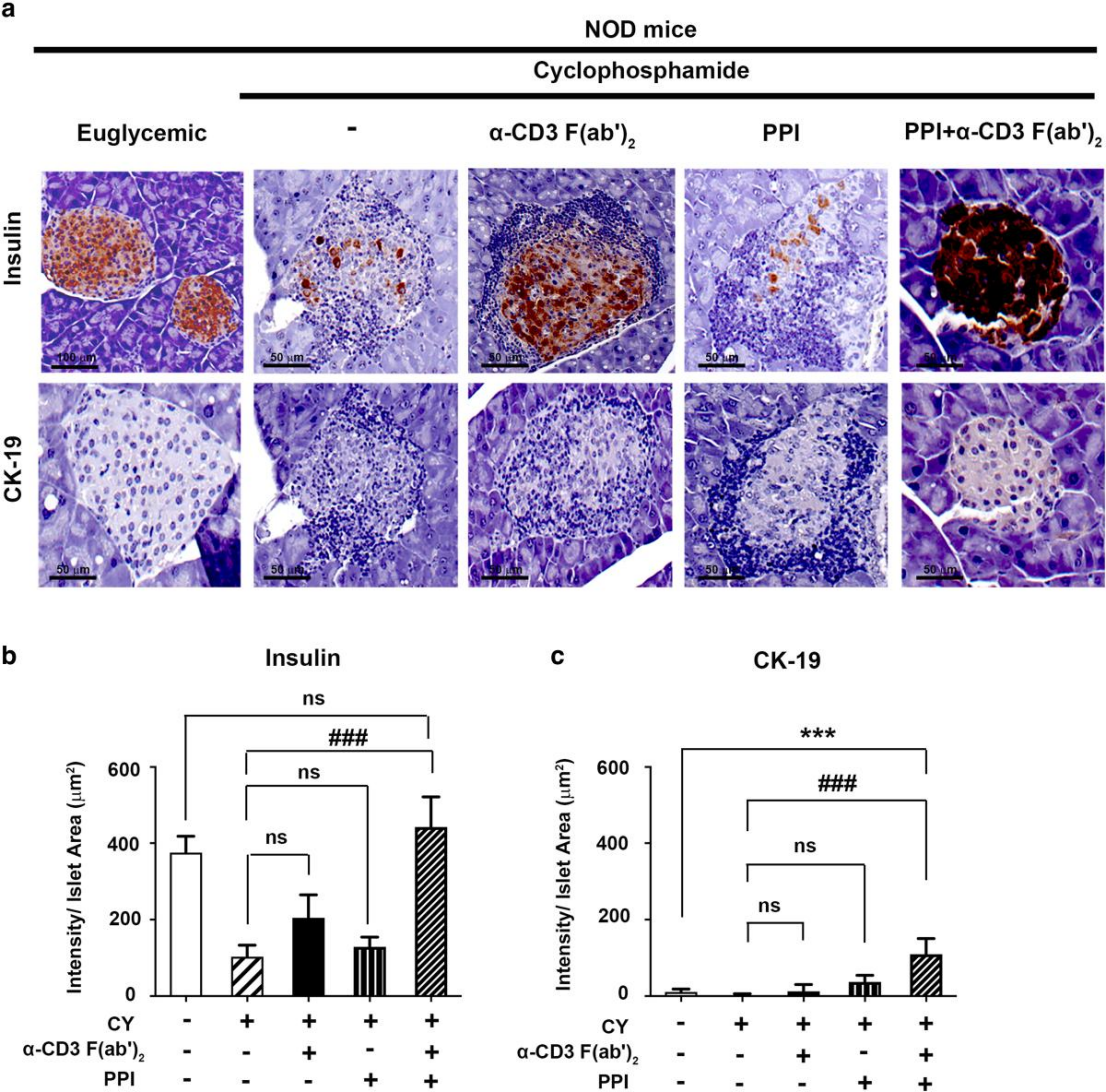
Insulin and CK-19 immunohistochemistry of pancreatic sections from CY-NOD mice treated with anti-CD3 F(ab’)_2_ and/or omeprazole. CY-NOD mice received anti-CD3 F(ab’)_2_ (10 µg/mouse, intravenous) and/or omeprazole (40 mg/kg/day, oral gavage) (*n* = 3 per group). After treatment, pancreatic tissues were collected and processed into paraffin sections for immunohistochemistry (IHC) to assess insulin or cytokeratin-19 (CK-19) expression. Sections were stained with anti-insulin (FNab04338, FinTest) or anti-CK-19 (10712-1-AP, Proteintech). Brown staining indicates positive immunoreactivity. (a) Representative images are shown for each group. (b) Quantification of insulin across treatment groups. (c) Quantification of CK-19 across treatment groups. *P < 0.05, **P < 0.01, ***P < 0.001 vs euglycemic mice; ^#^*P* < 0.05, ^##^*P* < 0.01, ^###^*P* < 0.001 vs untreated diabetic controls.

Biomarkers expressed on newly formed β-cells, such as cytokeratin-19 (CK-19), were examined using immunohistochemistry (IHC) in pancreatic islets to determine the therapeutic effects of various treatments on β-cell regeneration. In diabetic islet cells, although some islets exhibited only moderate insulitis scores based on the HE staining results, very few cells seemed to retain insulin secretory function without CK-19 expression. After treatment with the anti-CD3 F(ab’)_2_ or PPI alone, insulin-positive cells remained limited in number in the islets. Interestingly, we hypothesized that mice treated with the PPI would present with CK-19-positive cells because PPIs have been reported to induce islet neogenesis, but few CK-19-positive cells were observed, which may be due to newly formed β-cells being easily and rapidly eliminated by the autoimmune system. However, in mice treated with combination therapy, most cells in the islets retained insulin secretory function, and we observed increased levels of CK-19 expression in islet cells compared with euglycemic NOD mice, which indicated that combination therapy preserved insulin-positive cells and induced islet neogenesis (Fig. 4a–c).

### Combination therapy with an anti-CD3 F(ab’)_2_ and a PPI reverses diabetes progression in spontaneous-onset NOD mice

**Figure 5:**
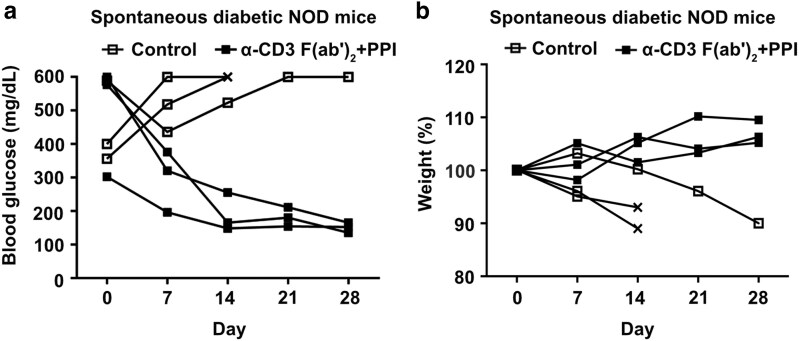
Blood glucose and body weight in spontaneous-onset diabetic NOD mice treated with anti-CD3 F(ab’)_2_ and PPI. Spontaneous-onset diabetic NOD mice (*n* = 3 per group) were treated after diabetes onset (blood glucose >300 mg/dL for >2 consecutive days) with anti-CD3 F(ab’)_2_ (10 µg/mouse, intravenous) plus omeprazole (40 mg/kg/day, oral gavage). (a) Blood glucose monitoring during the treatment period. (b) Body weight over time.

In the CY-NOD model, cyclophosphamide (CY) accelerates diabetes onset in part by reducing regulatory T cells and increasing the effector T-cell ratio. In contrast, spontaneous-onset NOD mice typically require a substantially longer period of autoimmune progression before hyperglycemia becomes evident [37]. To determine whether combination anti-CD3 F(ab’)_2_ and PPI therapy is effective in spontaneous-onset disease, NOD mice were treated only after hyperglycemia was detected. In untreated diabetic controls, blood glucose levels continued to rise without spontaneous remission, and one mouse died at week 3. In contrast, mice receiving the combination of anti-CD3 F(ab’)_2_ and PPI reverted to a euglycemic state and maintained stable body weight, whereas untreated controls showed an approximately 10% decrease (Fig. 5a and b).

**Figure 6:**
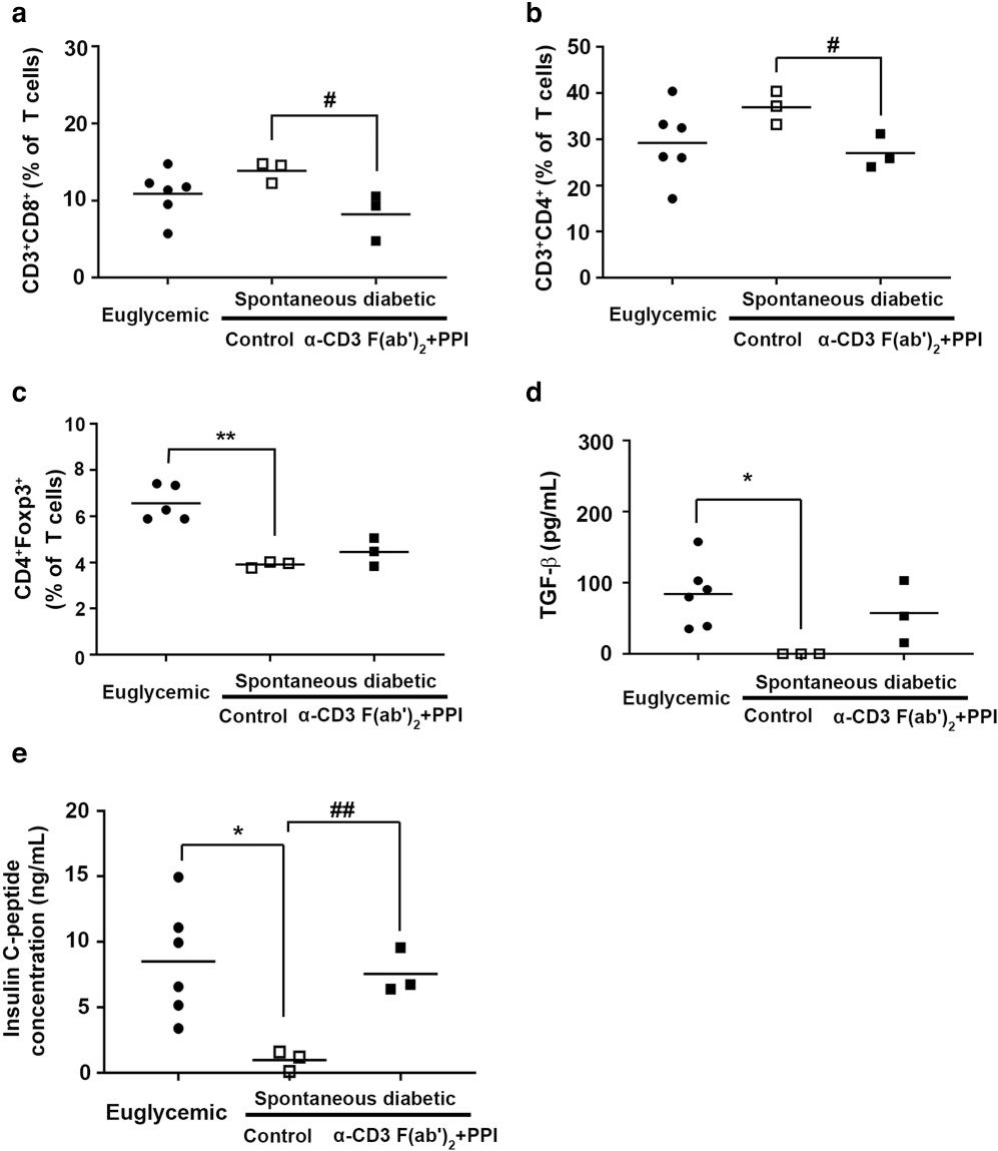
Splenic T-cell subsets, serum TGF-β and C-peptide in spontaneous-onset diabetic NOD mice treated with anti-CD3 F(ab’)_2_ and PPI. Spontaneous-onset diabetic NOD mice were treated with the combination of anti-CD3 F(ab’)_2_ and omeprazole or left untreated. Mice that survived to Day 28 after treatment were included for downstream analyses. (euglycemic controls, *n* = 6; untreated diabetic controls, *n* = 3; combination therapy, *n* = 3). Splenocytes were collected after treatment and analyzed by flow cytometry. (a) CD3^+^CD8^+^ T cells, (b) CD3^+^CD4^+^ T cells, and (c) regulatory T cells (Treg; CD3^+^CD4^+^Foxp3^+^). (d) Serum TGF-β and (e) C-peptide were measured by ELISA.*P < 0.05, **P < 0.01, ***P < 0.001 vs euglycemic mice; ^#^*P* < 0.05, ^##^*P* < 0.01, ^###^*P* < 0.001 vs untreated diabetic controls.

To further evaluate immune modulation in this model, splenocytes were collected after 4 weeks of treatment and analyzed by flow cytometry. Because spontaneous-onset diabetes required more than 10 weeks of observation in our experiments, the number of diabetic mice available for downstream immunophenotyping was limited. Nevertheless, spontaneous-onset diabetic mice exhibited increased frequencies of CD3^+^CD8^+^ and CD3^+^CD4^+^ T cells together with a reduced Treg frequency, consistent with the CY-NOD model (Fig. 2c–e). Following combination therapy, CD3^+^CD8^+^ and CD3^+^CD4^+^ T-cell frequencies were significantly reduced, and Treg frequency showed an increasing trend (Fig. 6a–c). In parallel, serum TGF-β levels were significantly reduced in spontaneous-onset diabetic mice compared with euglycemic controls, whereas combination therapy increased serum TGF-β levels toward those of euglycemic mice, with no statistically significant difference between the two groups and an upward trend relative to untreated diabetic mice (Fig. 6d).

Importantly, serum insulin C-peptide concentrations were markedly reduced in untreated diabetic mice (mean, 1.471 ng/mL) but increased ∼six-fold following combination therapy (mean, 8.391 ng/mL; Fig. 6e), indicating recovery of endogenous insulin secretory capacity. Collectively, these findings indicate that combination therapy with anti-CD3 F(ab’)_2_ and PPI is associated with diabetes reversal in spontaneous-onset NOD mice, characterized by restoration of euglycemia, reduced effector T-cell frequencies, and evidence of enhanced immunoregulation (including a trend toward increased Treg frequency and recovery of serum TGF-β). These results support further evaluation of this regimen as a potential non–insulin-dependent therapeutic approach for T1D.

## Discussion

In this study, we demonstrate that simultaneously inhibiting autoimmune dysregulation and supporting islet regeneration can preserve islet β-cell function and reverse T1D progression. We used a cyclophosphamide-accelerated NOD (CY-NOD) mouse model, together with intensive blood glucose monitoring, to approximate early-intervention therapy initiated while residual β-cell function was still present. Following treatment with a non–Fc-binding anti-CD3 F(ab’)_2_ combined with a proton pump inhibitor (PPI; omeprazole), hyperglycemic mice returned to the euglycemic range. Serum insulin C-peptide levels also increased after combination therapy, consistent with preserved endogenous insulin secretory function. Immune profiling showed a marked decrease in CD8^+^ T cells and an increase in Treg cells, together with increased TGF-β levels. In pancreatic sections, combination therapy reduced immune infiltration in islets and preserved a higher number of insulin-positive cells compared with control treatment. Moreover, we observed increased expression of the neogenesis-associated marker CK-19 in mice receiving combination therapy, consistent with an islet regenerative phenotype. We further evaluated the regimen in spontaneous-onset NOD mice, in which T1D progression occurs more slowly. Similar outcomes were observed after combination therapy in spontaneous-onset NOD mice, including reversal of hyperglycemia and recovery of insulin secretory function with no significant difference from healthy mice. Although Treg elevation did not reach statistical significance, an increasing trend was observed, and TGF-β levels increased. Together, these findings suggest that combining these two agents can reverse T1D progression and improve glycemic homeostasis.

Previously, T1D strategies focusing on anti-CD3 monotherapy have shown partial effectiveness in disease control. In a phase I/II trial, teplizumab treatment in early-onset T1D patients improved insulin secretion [26]. Teplizumab also delayed T1D progression in high-risk individuals in a phase II trial [26]. In another phase II/III trial, otelixizumab was reported to preserve β-cell function, as measured by C-peptide, with a more pronounced effect in patients with higher baseline C-peptide concentrations [23–25], indicating that anti-CD3 efficacy is closely related to residual β-cell function. Consequently, once diabetes has advanced to a stage with extensive β-cell loss, anti-CD3 therapy may only transiently preserve residual islet function rather than restore normoglycaemia [29]. Consistent with our findings, previous animal studies have also shown that anti-CD3 antibody monotherapy fails to restore normoglycemia in hyperglycemic NOD mice. In contrast, the combination of an anti-CD3 F(ab’)_2_ and a PPI showed a stronger disease-reversal phenotype in both spontaneous and CY-induced models in our study, resulting in restoration of euglycemia and improved insulin secretion, plausibly through immune regulation that limits autoimmune-mediated β-cell destruction while enabling islet regenerative processes.

In addition to modulation of autoimmunity, induction of β-cell regeneration is considered necessary for restoring insulin secretory function. Among putative regenerative mechanisms, gastrin-mediated pathways have been proposed to stimulate β-cell neogenesis; however, due to its short half-life, exogenous gastrin administration often fails to achieve sustained clinical benefit [38]. PPIs are among the most commonly used nondiabetic drugs that inhibit gastric acid secretion and can increase gastrin secretion [39]. Because PPIs do not directly lower blood glucose, they are not expected to cause hypoglycemia and have therefore been considered candidates for diabetes therapy [33]. Some studies have suggested that PPI treatment may benefit type 2 diabetes by supporting β-cell survival and increasing insulin secretion [40]. González-Oritz et al [41]. investigated pantoprazole and reported significant increases in late-phase and total insulin secretion. In a clinical trial in T1D, however, PPIs did not significantly improve insulin C-peptide levels, likely because ongoing autoimmunity eliminated newly formed β cells [42]. For late-stage diabetes, islet transplantation is a potential option, and intrahepatic islet transplantation has been used since 2000 to prevent severe complications; nevertheless, it is limited by donor availability [43]. In recent years, advances in stem cell and cell-therapy approaches (e.g. intravenous mesenchymal stem cells) have been explored to improve transplantation outcomes. In this context, our results support the rationale for combining a PPI with anti-CD3 F(ab’)_2_ to modulate autoimmunity and protect newly formed or recovering β cells, thereby improving glycemic control in NOD mice without the hypoglycemia risk associated with glucose-lowering drugs.

Protecting β cells and preserving endogenous insulin secretion is a central goal in T1D research, and multiple alternative approaches have been explored. In addition to intrahepatic transplantation [43], Navabi *et al*. treated NOD mice and streptozotocin-induced diabetic mice with stem cells combined with glucagon-like peptide 1 receptor agonists to promote islet implantation and support islet neogenesis [44]. Although stem cell-based strategies may provide a source of new islets for severe T1D, autoantibodies and autoimmunity can rapidly diminish efficacy [45]. Other immunosuppressive approaches include engineering *Lactococcus lactis* to produce cytokines such as interleukin (IL)-10 or IL-4 to reduce β-cell destruction [46–48]. While effective in early-onset animal models, translation to humans remains limited by biosafety and containment concerns, including risks of immune reactions, microbial containment, and long-term colonization by genetically modified microorganisms. In contrast, our strategy employs clinically used agents—an anti-CD3 F(ab’)_2_ and a PPI—to simultaneously modulate autoimmunity and support β-cell recovery, providing a potentially more feasible translational pathway.

In this study, we applied both the spontaneous NOD model and an accelerated model generated by administering cyclophosphamide to NOD mice [49]. Because Tregs are key regulators of T1D progression, cyclophosphamide is known to perturb Treg homeostasis and thereby accelerate diabetes onset in NOD mice [50], making the CY-NOD model well suited for probing the immunoregulatory component of our combination therapy. However, because CY-NOD progression is accelerated, we further evaluated the regimen in spontaneous-onset NOD mice to more comprehensively assess therapeutic effects in a model with a slower disease course.

The safety profile of this combination therapy warrants consideration. Regarding anti-CD3 F(ab’)_2_, the absence of the Fc domain reduces the risk of Fc receptor–mediated immune activation and cytokine release associated with conventional intact anti-CD3 antibodies [17, 51]. In clinical settings, short-course anti-CD3–based therapies for T1D, such as teplizumab, have shown a generally manageable safety profile, with common adverse events including transient lymphopenia, rash, leukopenia, headache, gastrointestinal symptoms, and mild infections [52]. Regarding PPI use, randomized controlled trial–based meta-analyses indicate no significant increase in the risk of pneumonia or enteric infections with PPI administration [53, 54]. While long-term PPI use has been associated with gut microbiota alterations in observational studies [55], the clinical relevance of this concern is likely limited in the context of short-course therapeutic use. Taken together, the agents employed in this combination regimen carry a manageable safety profile, though dedicated clinical studies will be necessary to confirm these findings in the human T1D setting. In conclusion, our study demonstrates that a combination of two clinically used drugs can promote immune regulation and support regenerative-associated features, resulting in reversal of T1D progression and preservation of islet function without insulin supplementation in NOD-based models. Importantly, this strategy is not expected to carry the hypoglycemia risk associated with glucose-lowering drugs. Future studies should define durability, optimal dosing/timing, and long-term safety to support translation.

## Supplementary Material

ltag017_Supplementary_Data

## Data Availability

The data supporting the findings of this study are included within the article and its online Supplementary material.
