# Supplementary material for: Combination of proton-pump inhibitor and anti-CD3 F(ab’)2 for islet neogenesis and immune modulation in type 1 diabetes
Source: Immunother Adv. 2026 Jul 22;6(1):ltag017. doi: 10.1093/immadv/ltag017 (PMC13426313; doi:10.1093/immadv/ltag017)
Supplement: ltag017_Supplementary_Data [file ltag017_supplementary_data.zip › Supplementary Figure Legends.docx]

**Supplementary Figure S1. Body weight trajectories and survival during anti-CD3 F(ab')₂ and PPI therapy in CY-NOD mice.**

Fifteen-week-old female NOD mice were rendered diabetic by cyclophosphamide (CY; 300 mg/kg, intraperitoneal) and treated with anti-CD3 F(ab')₂ (10 µg/mouse, intravenous) and/or omeprazole (40 mg/kg/day, oral gavage). Individual body weight trajectories are shown for (A) untreated diabetic controls (n=6), (B) anti-CD3 F(ab')₂ monotherapy (n=5), (C) omeprazole monotherapy (n=5), and (D) combination therapy (n=5). Each line represents an individual mouse. An “X” denotes the time point at which a mouse died during the monitoring period.
