# Supplementary figures and images for: Combination of proton-pump inhibitor and anti-CD3 F(ab’)2 for islet neogenesis and immune modulation in type 1 diabetes
Source: Immunother Adv. 2026 Jul 22;6(1):ltag017. doi: 10.1093/immadv/ltag017 (PMC13426313; doi:10.1093/immadv/ltag017)

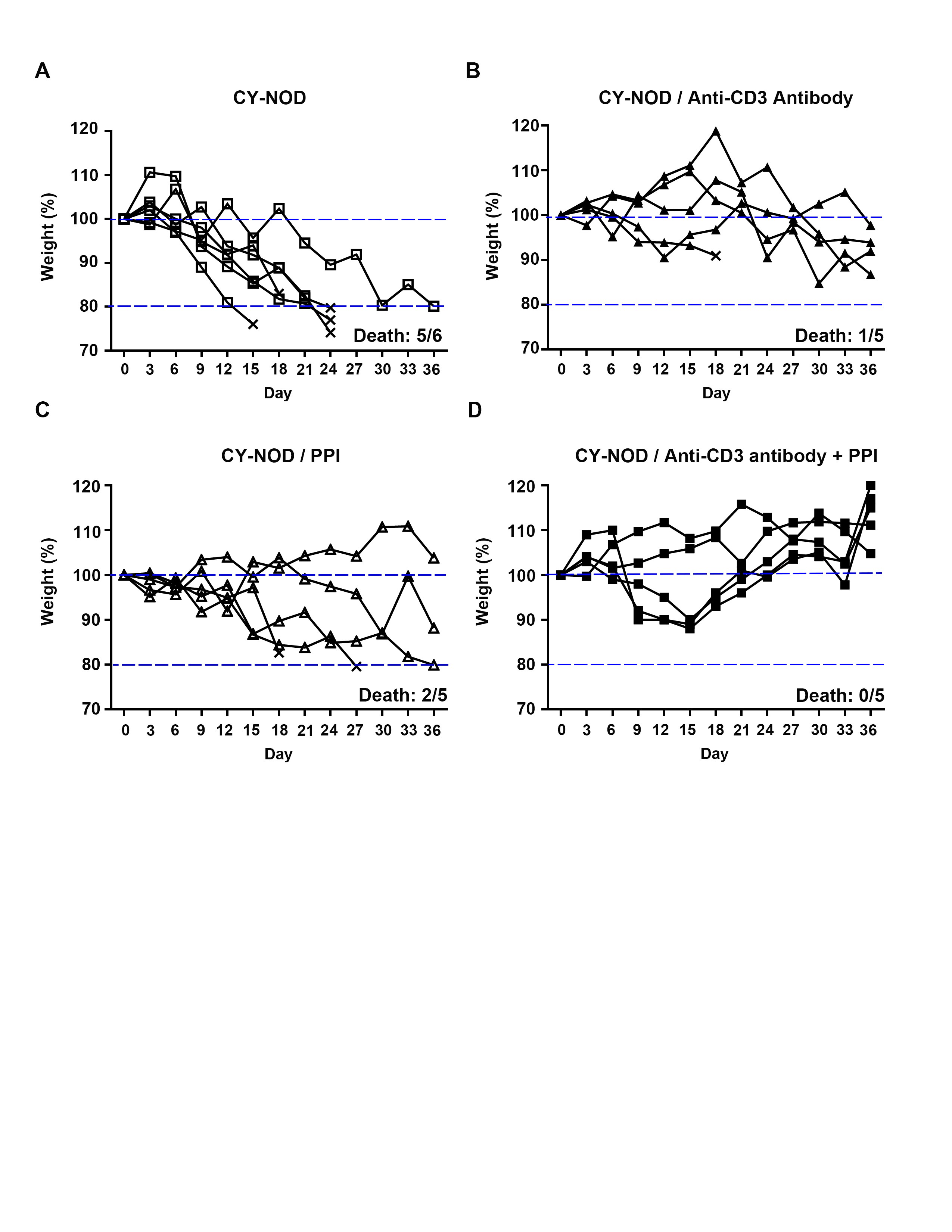

Supplement: ltag017_Supplementary_Data [file ltag017_supplementary_data.zip › sup1-ITA-2026-011.R1.tif]
